# Rare finding of ectopic pancreas in a cholecystectomy specimen: case report and literature review

**DOI:** 10.1093/jscr/rjae659

**Published:** 2024-10-21

**Authors:** Carolina Baz, Richard Nudotor, Ian Buseey, Kevin Stitely

**Affiliations:** Luminis Health Anne Arundel Medical Center, Department of Surgery, 2001 Medical Pkwy, Annapolis, MD, United States; Luminis Health Anne Arundel Medical Center, Department of Surgery, 2001 Medical Pkwy, Annapolis, MD, United States; Luminis Health Anne Arundel Medical Center, Department of Surgery, 2001 Medical Pkwy, Annapolis, MD, United States; Luminis Health Anne Arundel Medical Center, Department of Surgery, 2001 Medical Pkwy, Annapolis, MD, United States

**Keywords:** pancreatic tissue, ectopia, gallbladder, chronic cholecystitis, malignant transformation

## Abstract

Ectopic pancreatic tissue (EPT) is a rare condition in which pancreatic tissue is situated outside its normal position. It is commonly found in the stomach and small bowel, typically asymptomatic, and is usually discovered incidentally during histopathological analysis. Although fewer than 40 cases of ectopic pancreatic tissue in the gallbladder have been reported, its significance relies on the risk of malignant transformation, highlighting the need for a thorough pathological study. This case report describes the presence of EPT in the gallbladder incidentally found during the pathological examination following a laparoscopic cholecystectomy of a 37-year-old female due to chronic cholecystitis.

## Introduction

Heterotopic pancreatic tissue is an uncommon embryological abnormality defined as the presence of pancreatic tissue without any anatomic or vascular continuity with the pancreatic gland [1, 2]. The term originates from the fusion of two Greek words, “hetero,” which stands for “other,” and “topia,” which denotes “site,” recognizing the abnormal localization of the pancreatic cells [1, 3]. EPT is typically located along the gastrointestinal tract, with the stomach and small bowel being the most common sites. In contrast, the gallbladder (GB) is rarely affected. EPT can be diagnosed in any age group [3, 4], with a slightly higher prevalence in females in the case of EPT in the GB [5].

Most patients are asymptomatic, while others may have many nonspecific symptoms depending on the EPT’s location [4, 5]. When this tissue is attached to the GB, symptoms can mimic those experienced in acute or chronic cholecystitis [2]. Preoperative diagnosis of EPT in the GB can be challenging [2, 6], with most EPT cases being described as incidental histopathologic findings after cholecystectomy [2]. Given this heterotopic tissue can undergo the same pathological changes as the orthotopic one, the development of malignant transformation, among other etiologies, has been reported [4].

## Case presentation

**Figure 1:**
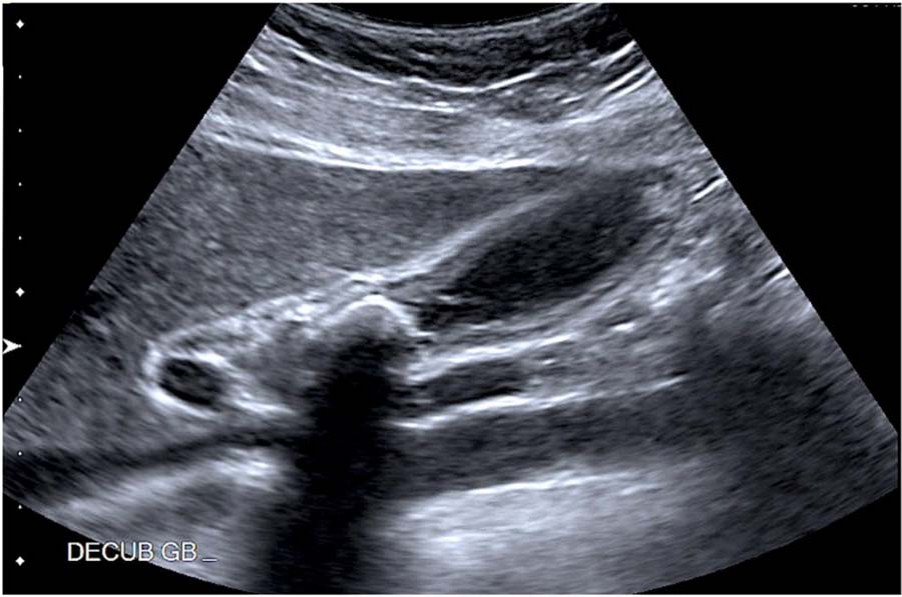
Abdominal ultrasound showing a 2.1 cm calcified gallstone in the gallbladder neck, and a thickened wall.

A 37-year-old female patient with a history of inflammatory bowel disease, gastroesophageal reflux disease, and a previous cesarean section was referred to the clinic for recurrent episodes of biliary colic. Her symptoms included right upper quadrant pain, along with nausea and vomiting. An abdominal ultrasound revealed a 2.1 cm gallstone in the GB neck and wall thickening. There was no dilation of the biliary tree, and the pancreas appeared normal (Fig. 1). Laboratory results did not show abnormalities, and the physical examination was unremarkable.

**Figure 2:**
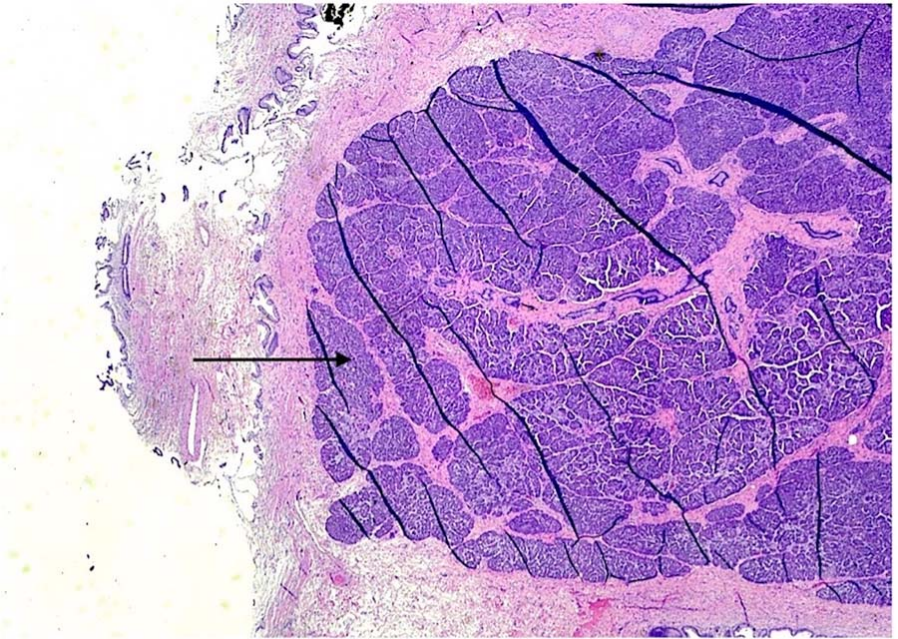
H & E stain 20×: Ectopic pancreatic parenchyma, composed of ductal and acinar components (black arrow).

**Figure 3:**
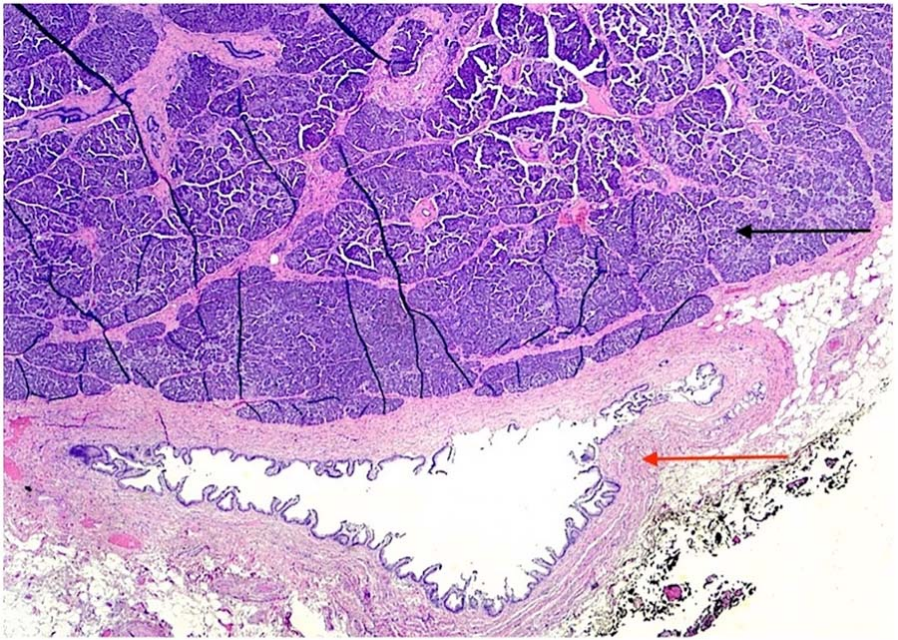
H & E stain 20×: Ectopic pancreatic parenchyma (black arrow), present in the gallbladder wall, composed of mucosa with underlying muscularis and adventitia (red arrow).

The patient underwent an elective laparoscopic cholecystectomy. During the surgery, findings were consistent with chronic cholecystitis. No abnormalities were visible upon gross inspection. Postoperatively, the patient recovered well and was discharged on the same day. The macroscopic histopathological examination revealed an intact GB with pink serosa. The mucosa was yellow-red without focal lesions. The microscopy showed chronic cholecystitis and the presence of a 1 cm focal nodule in the GB wall compatible with ectopic pancreatic parenchyma containing only acinar and ductal components (Figs 2–3). There was no evidence of dysplasia or malignant transformation in the analyzed sections. Since no malignancy was found among the EPT, there was no need for further treatment.

## Discussion

**Figure 4:**
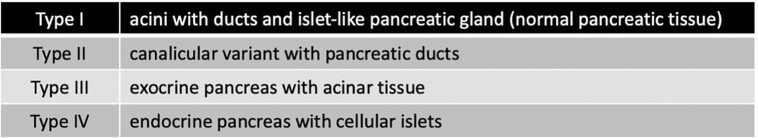
Classification system for pancreatic heterotopia by Gaspar Fuentes.

EPT was first described by Jean Schultz in 1727, but it wasn’t until 1909 that Von Heinrich provided the first classification based on pathological findings. Gaspar Fuentes then modified this classification in 1973 to include four types of EPT (Fig. 4) [1, 3]. This case corresponds to a type II EPT.

Various theories have been developed to explain the origin of this abnormal tissue. The most accepted one suggests that the EPC detaches from the primitive pancreatic gland during the rotation of the gastrointestinal tract in the embryogenic period [1]. Others propose that the abnormal location of the pancreatic tissue may be related to either the longitudinal growth of the intestine from the rudimentary pancreas or abnormalities in the signaling of the developing foregut endoderm tissue during embryogenesis [3].

EPT is rarely observed and is mainly found in the stomach (27.5%), duodenum (25.5%), and colon (15.9%) [3] with extraordinary locations, including the lung, mediastinum, and GB [5]. Its exact incidence remains unknown, but it has been identified in 2% of laparotomies and 0.5-13.7% of autopsies [2, 7]. The presence in the GB is even rarer, with less than 40 reported cases [1, 5]. Zhang *et al* [8] reviewed 184 cases of EPT, with only one case affecting the GB (0.5%). Similarly, a study from the Mayo Clinic involving 212 cases of EPT identified only one affecting the GB [9].

While EPT can be diagnosed at any age, it is typically found in people between 40 and 60. Most types show a male predominance with a ratio of 3:1 [3], except for the one in the GB. The higher prevalence in females is possibly due to the more frequent cholecystectomies related to cholelithiasis in women [4].

Patients with EPT are usually asymptomatic or may exhibit vague symptoms, such as pain, anorexia, or vomiting [2]. Nonetheless, symptoms can be more specific depending on the location of the tissue. Saeed *et al* [10] reported intussusception caused by EPT in the jejunum, along with other publications mentioning duodenal stenosis [11] or dysphagia [12] with duodenal or esophageal EPT. Colic-like symptoms have been described with EPT in the GB [4, 5, 13]. Some suggest biliary symptoms may be due to coexisting gallstones rather than EPT [5], while others [7] propose that the ectopic pancreatic tissue exocrine activity may cause pain and cholecystopathy, with or without accompanying gallstones.

Preoperative diagnosis of EPT in the GB can be challenging given its rarity, diffuse symptoms, and the limitations of diagnostic studies indistinguishing between ectopic pancreas and other GB pathologies such as polyps, adenoma, or neoplasia [1, 13]. The diagnosis usually occurs after surgery through histopathological analysis of the GB [1, 3, 4]. Macroscopically, this ectopic tissue can appear as an outward-growing mass, like polypoid lesions, or as a yellow-colored nodule [1, 3]. Approximately 55% of ectopic pancreas tissues are located in the gallbladder neck, with 73% found in the submucosa [2]. Histologically, it resembles the normal pancreas [2].

Being usually an incidental finding, its acknowledgment is important because, much like normal pancreatic tissue, EPT can potentially undergo different metaplastic and neoplastic changes [1, 7, 14]. Malignant transformation is rare, with a reported incidence between 0.7% to 1.8% [15]. Kaneko *et al* [15] found 52 cases of malignant transformation arising from EPT, including their own publication of adenocarcinoma originating from an ectopic pancreas in the duodenum. It is suggested that the pancreatic enzymes secreted from the EPT could negatively impact the GB mucosa, thus potentially causing GB cancer [1, 15].

The presence of EPT in the gallbladder is rare but significant and can cause nonspecific symptoms. It is not yet clear how EPT contributes to symptomatic gallstones or acute cholecystitis. However, it is important to consider EPT when diagnosing gallbladder disease, especially in cases with atypical symptoms or inconclusive imaging studies. Pathologists and surgeons should be aware of this condition to ensure proper diagnosis, management, and follow-up. Further research is needed to better understand the implications of ectopic pancreatic tissues and improve diagnostic accuracy.

This research received no specific grant from funding agencies in the public, commercial, or not-for-profit sectors.
